# Clinical and CT Features, Clinical Management, and Decision on Sport Eligibility of Professional Athletes with Congenital Coronary Anomalies: A Case Series Study

**DOI:** 10.3390/jcdd12010013

**Published:** 2024-12-31

**Authors:** Gianluca Guarnieri, Edoardo Conte, Davide Marchetti, Matteo Schillaci, Eleonora Melotti, Andrea Provera, Marco Doldi, Maria Rosaria Squeo, Antonio Pelliccia, Viviana Maestrini, Daniele Andreini

**Affiliations:** 1 Department of Biomedical and Clinical Sciences, University of Milan, 20122 Milan, Italy; gianluca.guarnieri@unimi.it; 2 Division of University Cardiology, IRCCS Ospedale Galeazzi Sant’Ambrogio, 20157 Milan, Italy; edoardo.conte86@gmail.com (E.C.); davide.marchetti@grupposandonato.it (D.M.); m.schillaci91@gmail.com (M.S.); eleonora.melotti@gmail.com (E.M.); andrea.provera@unimi.it (A.P.); doldi.mrc@gmail.com (M.D.); 3 Institute of Sport Medicine and Science, 00197 Rome, Italy; mariarosaria.squeo@coni.it (M.R.S.); antonio.pelliccia@coni.it (A.P.); viviana.maestrini@uniroma1.it (V.M.); 4 Department of Clinical, Internal, Anesthesiological and Cardiovascular Sciences, Sapienza University of Rome, 00185 Rome, Italy

**Keywords:** congenital coronary artery anomalies, myocardial bridge, sport elegibility, coronary fistula, cardiac arrest, athletes

## Abstract

Background: Congenital coronary artery anomalies (CAAs) are a significant cause of sudden cardiac death and a key factor in determining athletes’ eligibility for competitive sports. Their prevalence varies with diagnostic modalities and may present as asymptomatic or with life-threatening ischemic or arrhythmic events. This case series highlights the diverse manifestations of CAAs and the clinical approaches used to determine sports eligibility. Cases description: Five competitive athletes with different CAAs are presented. These cases include anomalous coronary origins, intramyocardial bridges, and coronary fistulas. Diagnostic tools, including coronary CT angiography (CCTA), cardiac magnetic resonance imaging (CMR), and stress tests, were essential in evaluating these anomalies and determining treatment strategies. In some cases, such as intramyocardial bridges, surgical intervention was necessary, while others required conservative management or exclusion from competitive sports. Conclusions: CAAs require individualized care based on risk stratification through advanced imaging techniques and functional assessment. Surgical interventions are reserved for high-risk anomalies, while others may be managed conservatively. Early detection and tailored management are crucial for ensuring athletes’ safety, and ongoing research is needed to optimize long-term outcomes.

## 1. Introduction

Congenital coronary artery anomalies (CAAs) represent one of the primary causes of cardiac arrest in young athletes and are also a leading reason for the denial of eligibility for competitive sports.

After an introduction about CAAs, we present a case series of five athletes, detailing their modes of presentation, diagnostic findings, and our decisions regarding the granting of eligibility for competitive sports. These decisions were made in accordance with the recommendations of the Cardiological Organizational Committee for Sports Eligibility (COCIS) [1,2].

### Background

The prevalence of CAAs remains unclear and varies significantly depending on the diagnostic method used. It ranges from 1% to 5% based on coronary angiography or autopsy studies and can be as high as 7.9% in studies utilizing coronary computed tomography angiography (CCTA) [3].

They are of various types and are commonly classified based on their anatomy:

- Anomalies of coronary artery origin: these occur when the coronary artery arises from an unusual or inappropriate location, such as outside of the appropriate aortic sinuses or from the pulmonary artery instead of the aorta. These anomalies can severely compromise blood flow to the myocardium, particularly during exercise, and are associated with an elevated risk of myocardial ischemia, heart failure, or sudden cardiac death [3];
- Anomalies of the coronary artery course: in these cases, the coronary artery course follows an abnormal path between major vascular structures, such as between the aorta and pulmonary artery. This abnormal course can lead to compression during periods of high blood flow, such as during intense physical activity, increasing the risk of ischemia, arrhythmias, or sudden cardiac arrest [4];
- Anomalies of coronary artery termination: these occur when the coronary artery terminates abnormally, such as draining into a cardiac chamber, pulmonary artery, or systemic veins. This type of anomaly can lead to inefficient oxygenation of the heart muscle and, in some cases, cause a coronary steal phenomenon, where blood is diverted from the myocardium, increasing the risk of ischemia and cardiac dysfunction, particularly during physical exertion [3].

All these abnormalities are summarized in Table 1.

The symptoms of these conditions can vary widely: from completely asymptomatic cases, where the diagnosis is often incidental, to sudden and fatal presentations with sudden cardiac death. In most cases, CAAs should be suspected when there are signs of ischemia in very young patients or when ischemic equivalents are detected during stress tests in athletes undergoing sports screening evaluations [3].

## 2. Diagnosis

The resting electrocardiogram (ECG) is usually normal in these patients but sometimes may already show abnormalities at rest, including ischemic repolarization changes such as ST-segment depression and T-wave inversion or flattening [5]. Echocardiography with color Doppler, especially in young individuals, should always aim to identify the origin of the epicardial coronary arteries [6]. When combined with a stress test, echocardiography can help determine whether known or undetected CAAs induce ischemia. However, in cases of high clinical suspicion for CAAs, coronary CT angiography (CCTA) is the gold standard for diagnosis and characterization of high-risk imaging features. This imaging modality is the most effective method for identifying coronary fistulas and intramyocardial bridges, and for evaluating coronary artery origins [7]. Cardiac magnetic resonance (CMR) is emerging as a valuable tool for diagnosing these conditions due to specialized sequences that allow for detailed evaluation of coronary artery origins [8]. Moreover, it allows for studies of cardiac chambers with volumes and dimensions. Additionally, with late gadolinium enhancement sequences, CMR can assess whether these anomalies are associated with myocardial fibrosis, providing further insights into the potential impact on cardiac function and risk stratification [9].

## 3. Case Presentation

### 3.1. Case 1: Anomaly of the Right Coronary Artery Origin Presented as Cardiac Arrest

**Figure 1:**
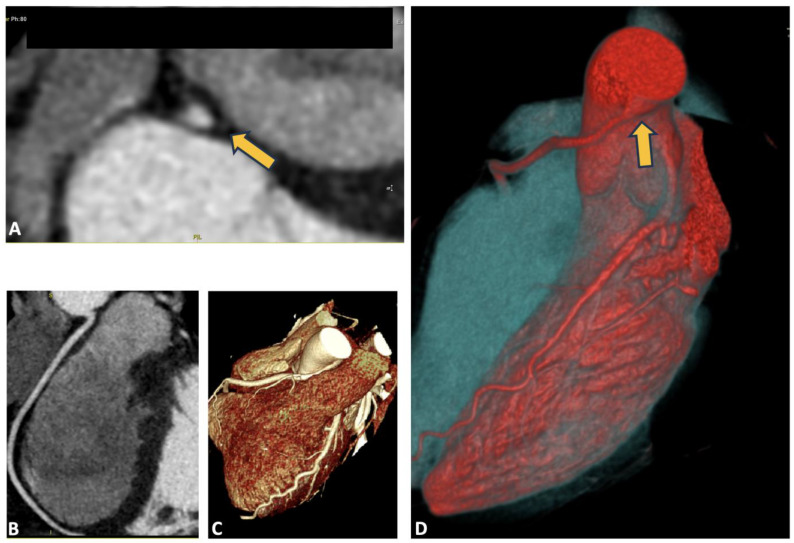
Anomaly of the right coronary artery from the left Valsalva sinus. In panel (**A**), the origin of the coronary artery is visible, showing an oval-shaped ostium, as indicated by the yellow arrow; panels (**B**,**C**) display the course of the vessel after its emergence from the left sinus; in panel (**D**), high take-off is observed, as indicated by the yellow arrow.

A 24-year-old patient, competitive cyclist without risk factors for ischemic heart disease, experienced a cardiac arrest due to ventricular fibrillation during competition. Cardiopulmonary resuscitation was performed, and circulation was restored after five dual shocks. The ECG did not reveal any significant baseline abnormalities, and laboratory tests were within normal limits. Coronary angiography showed no significant stenosis; however, a noteworthy finding was the anomaly in the origin of the right coronary artery (ARCA). The CCTA is shown in Figure 1. The CCTA revealed a right coronary artery originating from the left Valsalva sinus with a hostile proximal stenosis of <50%, an oval-shaped ostium, and a minimal lumen area (MLA) of 4.1 mm^2^ and a high take-off, all features that classify this anomalous origin as moderate risk [3]. The CCTA scan is shown in Figure 1.

Following a CMR that documented a linear region of late gadolinium enhancement in the inferior septum (non-ischemic pattern) and an electrophysiological study, which demonstrated reduced potentials in the septal area, the patient underwent implantation of a subcutaneous ICD, given the need for life-saving therapy and the absence of pacing requirements. The patient was no longer deemed eligible for competitive sports participation.

### 3.2. Case 2: Myocardial Bridge Presented as NSTEMI

**Figure 2:**
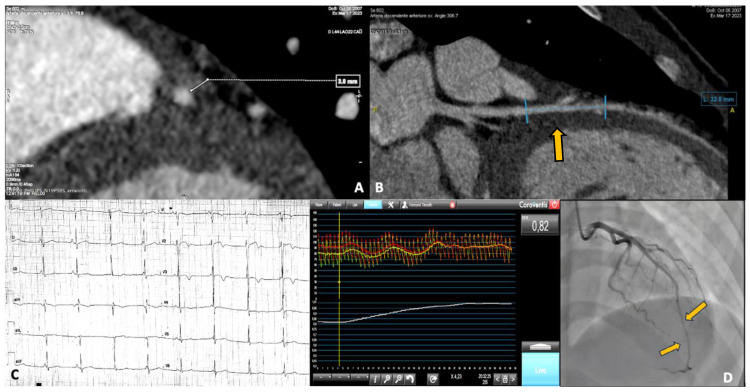
In panels (**A**,**B**), the CCTA images show the malignant features of the myocardial bridge, with a length > 25 mm and a depth > 2 mm. In panel (**C**), the anterolateral repolarization abnormalities present at baseline, before correction, are displayed. In panel (**D**), the coronary angiographic study shows the presence of the bridge at the mid-distal LAD, along with the functional assessment, with an RFR value of 0.82. An RFR of 0.82 indicates a hemodynamically significant and severe coronary stenosis.

A 15-year-old male competitive soccer player presented to the emergency department with chest pain following a soccer match. He was diagnosed with NSTEMI, showing anterolateral repolarization abnormalities with flat T waves and elevated cardiac enzymes. CMR was unremarkable, while coronary angiography revealed the presence of a myocardial bridge involving the mid LAD artery. A subsequent CCTA confirmed a severe myocardial bridge, measuring 30 mm in length and 3 mm in depth. All the investigations on this patient are shown in Figure 2. Panels A and B show the images from the CCTA. Panel C displays the repolarization changes observed on the electrocardiogram (ECG). Panel D presents the myocardial bridge as seen during coronary angiography, along with functional assessment findings.

The patient, given his young age and presentation as an acute coronary syndrome, underwent surgical correction of the myocardial bridge. Four months after intervention, following a CMR that did not show areas of myocardial fibrosis and the absence of an arrhythmic substrate, the patient was granted eligibility for competitive sports.

### 3.3. Case 3: Right Coronary Artery Originating from the Left Valsalva Sinus

A 33-year-old young woman, a highly trained competitive runner specializing in middle-distance events, presented to the cardiologist following sporadic episodes of palpitations; she reported no angina or dyspnea. A Holter ECG revealed the presence of 2550 premature ventricular beats (PVB) over a 24 h period, and the stress-ECG maximal test (300 W) was negative for inducible ischemia, with the presence of PVB at rest and also during exercise (isolated and couples). All PVBs reported at Holter and stress-ECG showed the same morphology (right bundle branch block), suggesting their origin from the inferior wall of the left ventricle.

**Figure 3:**
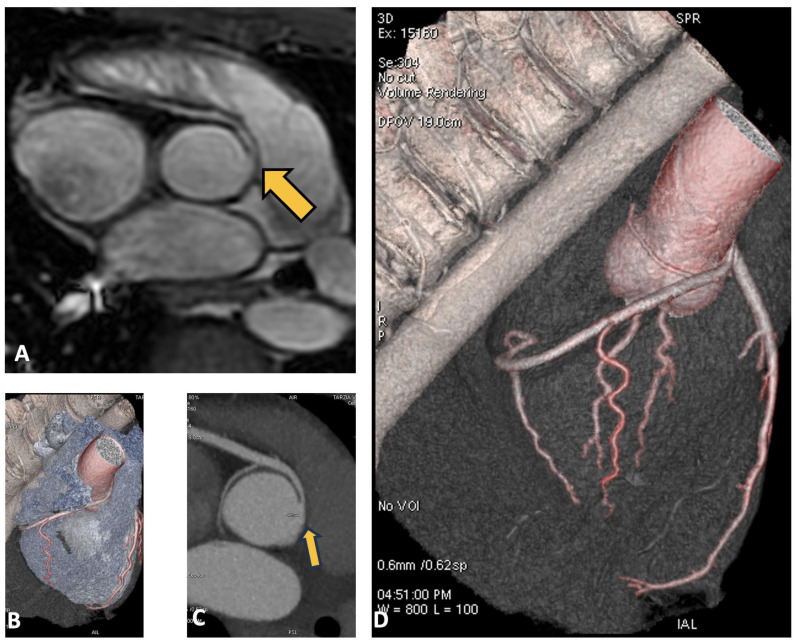
In panel (**A**), the cardiac MRI in the coronary study sequence shows the right coronary artery, indicated by the yellow arrow, originating from the left Valsalva sinus. In panels (**B**–**D**), similar images from the CCTA are shown, with the right coronary artery originating from the left sinus.

The patient subsequently underwent CMR, which showed normal biventricular volumes and function, with no evidence of myocardial fibrosis. However, specific sequences aimed at studying the origin of the coronary vessels identified the presence of a right coronary artery originating from the left Valsalva sinus. To complete the diagnostic assessment, the patient then underwent CCTA, which confirmed the diagnosis and revealed high-risk features, including the presence of a proximal intramural course and a “slit-like” ostial appearance, as illustrated in Figure 3.

Due to the aforementioned characteristics, the patient was not granted clearance for competitive sports participation.

### 3.4. Case 4: Coronary Fistula

A 27-year-old female elite volleyball player reported new onset of sporadic episodes of precordial pain during intense training, which culminated in syncope on two occasions and near-syncope once. On one of these occasions, she was taken to the hospital, where an emergency coronary angiography revealed a condition described as multiple coronary fistulas originating from the LAD.

The rest ECG was normal, and the echocardiogram did not show any contraction abnormalities.

CCTA showed two fistulas originating from two points in the LAD, which, however, do not supply the left ventricle but instead the right ventricle. Furthermore, the fistulous tract does not merely extend to the ventricular wall but opens into the cavity itself, resulting in a shunt condition.

**Figure 4:**
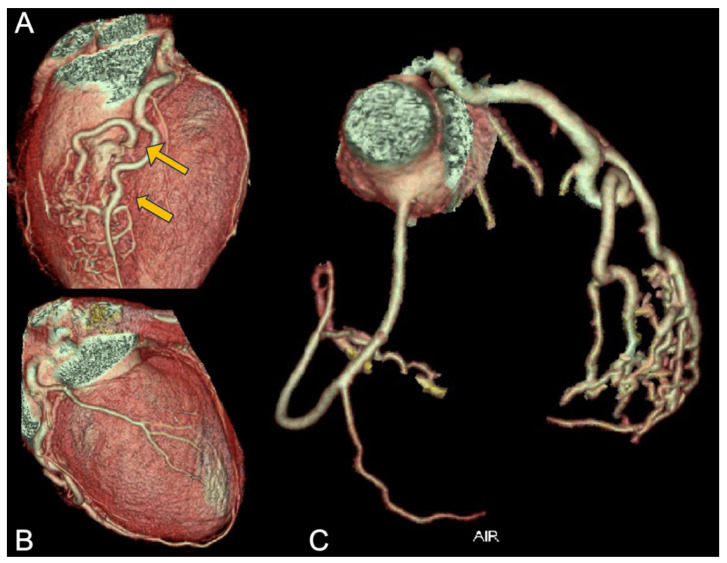
CCTA images of the patient from case 4 with coronary fistula; the two yellow arrows indicate the two points where the fistulous tracts begin, which then give rise to the coronary malformation.

The patient was referred to a tertiary care center, where she underwent a stress CMR with dipyridamole, revealing a significant anterior perfusion deficit. The surgeon opted not to operate, deeming it practically a form of arteriovenous malformation and technically inoperable. The CCTA is shown in Figure 4.

As a result, the patient was declared unfit for competitive sports. After several months, the patient stopped playing and is currently asymptomatic, having experienced symptoms only during intense physical exertion.

### 3.5. Case 5: Common Origin of Coronary Arteries from Left Valsalva Sinus with Interarterial Course

A 31-year-old patient, a competitive endurance athlete, completely asymptomatic, with a finding of PVBs presented for a follow-up visit for competitive sports eligibility.

The athlete reported no symptoms of angina, dyspnea, palpitations, or syncope either at rest or during exertion. The family history was negative for sudden juvenile death and cardiomyopathies, and the personal medical history did not indicate any conditions of internal medicine or cardiovascular interest. The athlete was not undergoing any therapy.

The baseline ECG showed sinus rhythm at a heart rate of 72 bpm, balanced electrical axis, AV conduction within normal limits, incomplete RBBB, and early repolarization. QTc was within normal limits.

An exercise stress test revealed extrasystoles at the peak of exertion, with the occurrence of two extrasystoles of the same morphology (LBBB and superior axis). Additionally, a 24 h Holter ECG recorded four ventricular extrasystoles of different morphology (LBBB with superior axis and LBBB with inferior axis), three of which occurred during a training session.

The echocardiogram with color Doppler showed normal biventricular systolic function and the absence of significant valvular disease.

In light of the arrhythmia, though rare but polymorphic, induced, and reproducible by exertion, the athlete underwent a CMR with contrast, which did not reveal any abnormalities in the morpho-functional evaluation of the cardiac chambers or in tissue characterization. However, suspicion was raised regarding an anomalous origin of the right coronary artery and an intramyocardial bridge of the left anterior descending artery.

**Figure 5:**
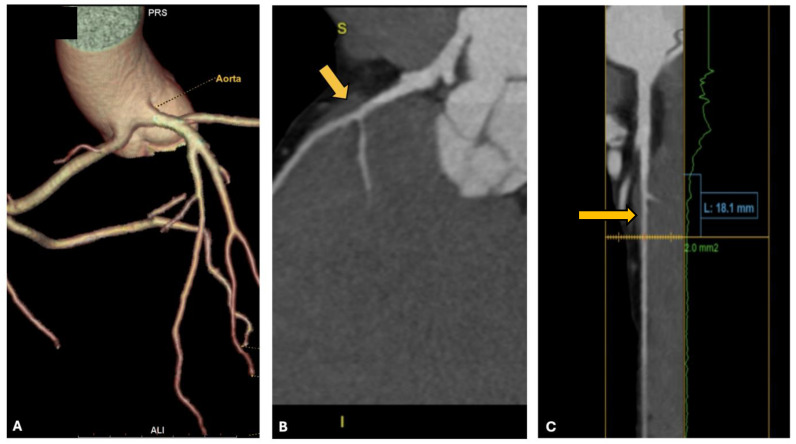
CCTA of the patient from case 5. In panel (**A**), the common origin of the right and left coronary arteries from the left Valsalva sinus is shown; in panels (**B**,**C**), the yellow arrows indicate the presence of the intramyocardial bridge on the LAD.

Consequently, the athlete underwent CCTA, which confirmed the presence of an anomalous origin of the right coronary artery and the common trunk just above the junction between the right and left coronary sinuses, with an interarterial course of both vessels, but no signs of intramural course (absence of “slit-like” ostium or reduction in diameter at the origin and proximal segment). It is shown in Figure 5. Furthermore, the presence of a very short first segment of the LAD was noted, 2 mm deep and 18 mm long, with the first diagonal branch originating very proximally and a myocardial bridge over the second segment.

The patient was declared ineligible for competitive sports.

## 4. Discussion

As described above, CAAs are rare conditions, but their incidence is increasing, partly due to the use of CCTA, which enables diagnosis even in patients who do not undergo invasive coronary angiography. Among CAAs, the most common are anomalies of origin and intramyocardial courses. All the cases with their final decisions on sport eligibility are summarized in Table 2.

### 4.1. Intramyocardial Bridge

IMBs are congenital anomalies in which a segment of a coronary artery, typically the left anterior descending artery, is tunneled within the myocardium rather than running along the epicardial surface. The prevalence of this condition varies significantly depending on the imaging modality used for diagnosis. Data show a prevalence of around 6% with coronary angiography, increasing to as high as 22% when diagnosed using CCTA.

During systole, the myocardial contraction can compress the bridged segment of the artery, potentially leading to transient reductions in coronary blood flow. Myocardial perfusion occurs during the diastolic phase of the cardiac cycle, which is why intramyocardial bridges were long considered benign conditions. However, intracoronary imaging techniques, such as intravascular ultrasound (IVUS), have shown that after the systolic phase, there is also delayed relaxation of the bridged segment during diastole [10]. This delayed relaxation can impair coronary blood flow, particularly under conditions of increased demand, challenging the traditionally benign view of this condition [10]. Additionally, coronary blood flow can be altered due to coronary steal associated with the Venturi effect; indeed, during systole, the bridge compresses the artery, accelerating blood flow through the compressed segment [11]. This Venturi effect reduces downstream pressure, diverting blood away from collateral branches, which can exacerbate myocardial ischemia [12].

Superficial intramyocardial bridges, defined as those less than 2 mm deep, are typically asymptomatic in most cases. In contrast, deep bridges—those deeper than 2 mm—or those longer than 25 mm are more likely to cause ischemic symptoms, as they exert greater compression on the coronary artery and have a more pronounced impact on coronary blood flow [13].

The COCIS recommends granting competitive sports eligibility to athletes with an intramyocardial bridge discovered incidentally through a CCTA performed for an inconclusive/positive stress test or other reasons, provided that no inducible ischemia is detected (on treadmill testing, physical stress echocardiography, myocardial perfusion scintigraphy, or coronary angiography); athletes with a significant intramyocardial bridge (long and deep) who show signs of exercise-induced ischemia should be excluded from competitive sports. Their eligibility can be re-evaluated after 6 months if they undergo surgical correction and remain symptom-free, with assessments carried out at specialized centers. Indeed, the patient in case 2, after undergoing surgical correction of the myocardial bridge, returned to competitive play. Clearance for athletic participation was granted, in accordance with the guidelines, after a period of at least 6 months without symptoms, without any arrhythmic events, and in the absence of evidence of fibrosis on CMR.

Asymptomatic cases do not require treatment. For symptomatic cases, medical therapy is the first choice, typically involving beta-blockers to reduce myocardial oxygen consumption and prolong diastole. If medical therapy is ineffective, stenting the intramyocardial bridge segment may be considered to maintain lumen during systole and diastole, though this approach is not universally endorsed. The preferred treatment for refractory cases is surgical resection of the myocardial segment above the bridge [14].

### 4.2. Abnormalities of the Origin of Coronary Arteries

CAAs of origin include coronary arteries that arise from the aorta but from an incorrect sinus of Valsalva, as well as those that originate from the pulmonary artery. In cases where coronary arteries originate from the opposite sinus, it is important to also assess their course. The potential pathways include retroaortic, interarterial, subpulmonic, prepulmonic, or retrocardiac routes, each of which may have different clinical implications [15].

CCTA is the gold standard for evaluating not only the course of coronary anomalies but also the features that contribute to their malignancy [16]. It allows for detailed visualization of the coronary ostia, determining whether they are separate or shared. Additionally, it helps assess the proximal segment, identifying dangerous characteristics such as proximal hypoplasia or a “slit-like” appearance, both of which are high-risk features. Conversely, a well-developed proximal segment indicates a lower risk. Moreover, CCTA can accurately measure the take-off angles of coronary vessels from the sinuses of Valsalva. Therefore, sharp angles are linked to higher risk.

Prepulmonic courses are usually benign because they often feature right-angle take-offs and follow an extramural path. A retroaortic course typically involves the circumflex artery originating from the right sinus of Valsalva, either independently from the right coronary artery or with a shared ostium. After originating from the right side, the artery follows a retroaortic path, passing between the atrium and the aorta, before reaching the left atrioventricular groove [15].

The interarterial course involves the anomalous origin of a coronary artery from the opposite sinus of Valsalva—for example, the right coronary artery from the left sinus or the left coronary artery from the right sinus. The primary concern with this anomaly is not the interarterial course itself, as pulmonary hypertension is necessary for significant compression between vessels (which is rare given the lower pressure in the pulmonary artery). The real issue arises when a subset of these anomalies has a proximal intramural course, where the artery runs within the aortic wall, either within the tunica media or between the media and adventitia [17]. This congenital defect prevents the artery from developing properly, leaving the intramural segment weakened and hypoplastic. The coronary ostium often takes on a “slit-like” or “flute beak” shape, which contributes to ischemia, independent of atherosclerosis. These anomalies are frequently associated with a very sharp take-off angle, typically less than 45 degrees, further increasing the risk of ischemic event [18].

A subpulmonic course involves a coronary artery passing beneath the pulmonary artery. This is typically seen when the left coronary artery arises abnormally from the right sinus of Valsalva, traveling under the pulmonary artery before reaching its normal destination. While generally considered less risky compared to other anomalous courses, it can still pose a risk depending on factors like the angle of origin and vessel compression [15].

The COCIS guidelines allow young athletes with an anomalous circumflex artery from the right sinus or right coronary artery (retroaortic course) to participate in sports if coronary CT and stress tests show no ischemia.

An anomalous origin of the left coronary artery from the right sinus with an interarterial or intramural course should lead to exclusion from all competitive sports and a strongly recommendation for surgical correction in symptomatic individuals. For asymptomatic individuals (discovered incidentally), opinions vary, but most experts advocate for surgery due to the significant risk of myocardial ischemia and sudden cardiac death. If surgery is not performed, it is strongly advised to avoid high-intensity physical activities and consider beta-blocker therapy.

Exclusion from competitive sports is recommended for athletes with symptomatic right coronary artery anomalies originating from the left sinus with an interarterial course. It is also advised for asymptomatic individuals with high-risk anatomical features, like a “slit-like” ostium, an intramural course, or proximal hypoplasia. Indeed, our patient from case 5 was declared eligible because he was asymptomatic, and the only malignant condition was an acute take-off angle; however, the other characteristics were low-risk, and most importantly, we documented the absence of ischemia during stress tests. However, we have also illustrated the opposite case: the athlete from case 3 had a negative stress test for inducible ischemia but was declared ineligible due to malignant characteristics observed on CCTA, including an interarterial course and a slit-like ostium, as well as being symptomatic with palpitations.

A statement from the task force of American heart Association in case of anomalous origin of the left coronary artery from the right sinus of Valsalva suggests restricting all athletes from all competitive sports, with the possible exception of low-intensity class sports, especially if the anomalous artery passes between the aorta and the pulmonary artery (Class III; Level of Evidence B), before surgical repair; athletes with an anomalous origin of the right coronary artery from the left sinus of Valsalva should undergo an exercise stress test, and if they are asymptomatic with a negative test result, they may be allowed to compete after being fully informed about the risks and limitations of the test’s accuracy [19].

Athletes with a high take-off origin who are asymptomatic and have no evidence of ischemia or arrhythmias on stress testing or imaging may be deemed eligible for competitive sports participation, according to ESC 2020 guidelines; however, if ischemia or symptoms are present, further evaluation and intervention are warranted prior to clearance for high-intensity physical activities [20].

Case 1 was likely the most straightforward in terms of management. In addition to having clear malignant characteristics on CCTA, the clinical presentation began with cardiac arrest, and the patient subsequently underwent ICD implantation. Therefore, the patient was declared ineligible.

Cases 1 and 3 thus align consistently with the COCIS guidelines.

Nevertheless, there are also grey areas where the COCIS has not provided clear guidelines, partly due to the lack of strong scientific evidence. Indeed, the athlete of case 5 presents with a unique anomaly where both coronary arteries arise from a single ostium in the left Valsalva sinus, without malignant anatomical features but with a short interarterial course that is considered non-benign. This case is not explicitly covered in the current sports eligibility guidelines of COCIS 2023, which only address similar anomalies under different conditions. Additionally, the athlete has an intramyocardial bridge. Moreover, the athlete also presents with rare but polymorphic ventricular extrasystoles that are reproducible during physical exertion. At present, it is not possible to definitively attribute the arrhythmias to the subject’s coronary anomalies; however, their characteristics, such as polymorphism and inducibility by physical exertion, classify them as non-benign. This factor is considered an additional risk element. Therefore, in light of these considerations, the Commission deems the athlete ineligible to continue the specific competitive activity.

### 4.3. Coronary Artery Fistula

Coronary fistulas are rare conditions affecting approximately 0.1% of the population. In most cases, they are congenital, although they can also form as a result of intracardiac device implantation procedures [21]. The fistulous tract may connect a coronary artery to another vessel or a cardiac chamber. In the majority of cases, patients are asymptomatic, though these fistulas can occasionally lead to life-threatening complications.

In fact, if the area supplied by a coronary fistula is not perfused by other epicardial coronary arteries, coronary steal syndrome can occur, leading to ischemic episodes at rest or during exertion, potentially resulting in angina pectoris. Additionally, if an epicardial coronary artery drains into a cardiac chamber, causing a shunt—especially in cases where the fistula communicates with the atria or the right ventricle—this can lead to dilation of the affected chamber and subsequent heart failure [22].

As for treatment, it is often conservative. There are limited published data and no clear guidelines regarding surgical or interventional therapy. However, Al-Hijji, in a study published in *JACC Interventions* in 2021 [21], recommends closing the fistula if it is medium or large in size and symptomatic, particularly in the presence of ischemia in the area supplied by the affected artery, if there are life-threatening arrhythmias due to the fistula, or if cardiac dysfunction or dilation of the involved chamber occurs.

Our patient in case 4 exhibited the classic symptoms of a severe fistula, including exertional angina. The presence of ischemia was later confirmed by the finding of a contractility deficit on stress MRI. However, the anatomical configuration of the fistula did not allow for surgical intervention, so a conservative treatment approach was chosen.

The COCIS states that for small, asymptomatic coronary fistulas that do not show signs of ischemia, arrhythmias, or heart dysfunction, athletes can be cleared to participate in all sports, as long as they have regular heart check-ups. For larger fistulas that cause significant shunting, myocardial ischemia, arrhythmias, or heart dysfunction, athletes should not be cleared for sports. In such cases, closing the fistula—through surgery or a minimally invasive procedure—is recommended. The decision to allow an athlete to return to sports after the procedure should only be made by highly specialized centers.

Our patient, due to all the characteristics described above, was not declared eligible according to the COCIS guidelines.

Nonetheless, with the cessation of intense physical activity, the patient has since remained asymptomatic.

## 5. Phycological Consequences of Disqualification

The psychological effects of being disqualified from sports participation for medical reasons can be deeply impactful, particularly for athletes who closely associate their identity and self-worth with their athletic performance. Disqualification can trigger intense feelings of frustration, sadness, and loss, as athletes may view the decision as the end of their aspirations, social standing, or career prospects. Research by Lavallee et al. found that athletes in such situations often experience emotional reactions similar to the stages of grief, including denial, anger, and eventual acceptance. Similarly [23], Wiese-Bjornstal et al. identified increased risks of anxiety, depression, and social withdrawal among athletes who face career-altering medical setbacks [24].

The psychological toll can be especially severe for young or elite athletes, for whom sport often serves as a core aspect of their identity and social framework. Losing the ability to participate may strain relationships with teammates and coaches, heighten feelings of isolation, and remove a key source of stress relief. For many athletes, the sudden absence of physical activity as a coping mechanism can intensify emotional challenges.

To address these issues, it is essential to provide comprehensive psychological support alongside medical care. Studies like those by Brewer and Petitpas highlight the value of mental health interventions, such as therapy, peer support groups, and structured career transition programs, to help athletes process the emotional impact of disqualification [25]. Clear communication about the medical reasons for the decision, coupled with encouragement to explore new roles within the sports world—such as coaching or mentoring—can foster a sense of purpose and connection. These approaches not only help athletes adapt to their new circumstances but also promote long-term mental well-being and resilience.

## 6. Conclusions

CAAs are a critical consideration in sports cardiology due to their association with ischemic events and sudden cardiac death, particularly in young athletes. This case series highlights the diverse presentation of CAAs, including anomalous coronary origins, myocardial bridges, and coronary fistulas, demonstrating the importance of comprehensive diagnostic approaches such as CCTA and CMR.

Risk stratification, guided by functional assessments like stress tests and invasive angiography, plays a pivotal role in determining sports eligibility. While surgical intervention is necessary in high-risk cases showing ischemic potential, conservative management may suffice for less severe anomalies, enabling athletes to safely continue competitive activities with proper monitoring.

The findings emphasize the need for early detection and tailored management of CAAs, reinforcing the importance of individualized care. Ongoing research is essential to improve long-term outcomes and refine guidelines for athletes with these rare but potentially life-threatening conditions.

## Figures and Tables

**Table 1:** Classification of congenital coronary abnormalities.

Type of Anomaly	Variant	Type	Treatment	Case Example
Anomalies of Origin	Coronaries originating from pulmonary artery	- left main coronary artery from the pulmonary artery - right coronary artery from the pulmonary artery - circumflex coronary artery from the pulmonary artery - both coronary arteries from the pulmonary artery	Surgical intervention, depending on symptoms and ischemia	None
	Anomalous aortic origin of the coronaries	- left main coronary artery from the right aortic sinus of Valsalva - right coronary artery from the left aortic sinus of Valsalva - left anterior descending coronary artery from the right aortic sinus of Valsalva - left anterior descending coronary artery from the right coronary artery - circumflex coronary artery from the right aortic sinus of Valsalva - circumflex coronary artery from the right coronary artery	Surgical revascularization or reimplantation if symptomatic	1, 3, 5
Anomalies of Course	Intramyocardial bridge	- superficial - long and deep: length > 25 mm and 2 mm deep	Beta-blockers, lifestyle modification, surgery if severe	2
	Coronary aneurysm	congenital	Surgical repair or monitoring based on symptoms	None
Anomalies of Termination	Coronary fistula	- congenital - acquired: after procedure of cardiac device implantation	Conservative of surgical ligation/catheter closure if very symptomatic	4

**Table 2:** Presentation mode and decision on sports eligibility in our cases.

Case	Age	Sport Type	Coronary Anomalies	Presentation	Sport Elegibility
1	24	Cycling	Anomalous right coronary artery	Cardiac arrest during exercise	No
2	15	Soccer	Myocardial bridge	NSTEMI	Yes
3	33	Running	Right coronary artery from left Valsalva sinus	Palpitations	No
4	27	Volleyball	Coronary fistula	Syncope	No
5	31	Triathlon	Anomalous right coronary artery, myocardial bridge	Asymptomatic, ventricular extrasystoles	No

## Data Availability

Available data are reported in the paper.
